# Partial versus radical nephrectomy for pT3a renal cell carcinoma: a systematic review and meta-analysis

**DOI:** 10.1007/s11701-026-03384-8

**Published:** 2026-04-21

**Authors:** Filippo Gavi, Francesco Rossi, Daniele Fettucciari, Giuseppe Pallotta, Antonio Silvestri, Cristina Carerj, Vincenzo Cavarra, Domenico Sanesi, Francesco Pio Bizzarri, Marco Montesi, Simone Assumma, Enrico Panio, Pierluigi Russo, Mauro Ragonese, Nazario Foschi, Filippo Turri, Maria Chiara Sighinolfi, Bernardo Rocco

**Affiliations:** 1 Department of Urology, Fondazione Policlinico Universitario Agostino Gemelli IRCSS, Rome, Italy; 2 Università Cattolica del Sacro Cuore, Rome, Italy; 3 Department of Urology, Ospedale San Filippo Neri, Rome, Italy; 4 Department of Urology, Isola Tiberina - Gemelli Isola Hospital, Rome, Italy

**Keywords:** Kidney, Renal cancer, Partial nephrectomy, Radical nephrectomy, Locally advanced, Surgery

## Abstract

The surgical management of pathologic T3a (pT3a) renal cell carcinoma (RCC) remains contentious due to the lack of high-level evidence guiding the choice between partial nephrectomy (PN) and radical nephrectomy (RN). This systematic review and meta-analysis aims to evaluate whether PN represents a safe and effective alternative to RN. A comprehensive search of PubMed, Web of Science, and Scopus was conducted through July 2025. The study included comparative trials of adult pT3a RCC patients undergoing PN or RN, focusing on oncological, perioperative, and functional outcomes. Sixteen retrospective studies involving 34,304 patients (5878 PN; 28426 RN) were analyzed. There were no statistically significant differences between PN and RN regarding estimated blood loss, operative time, hospital stay, or major postoperative complications (Clavien-Dindo > 2). PN was associated with significantly better preservation of renal function (9.96; I^2^ 0%; *p* < 0.01), higher rate of positive surgical margins (0.82; I^2^ 49.2%; *p* < 0.01), lower tumor size (-2.6; I^2^ 99.4%; *p* < 0.01). PN is a safe and feasible alternative for pT3a RCC cases, providing superior functional preservation without compromising perioperative safety or oncological control.

**Supplementary Information:**

The online version contains supplementary material available at 10.1007/s11701-026-03384-8.

## Introduction

Renal cell carcinoma (RCC) accounts for approximately 2% to 3% of all adult malignancies [1]. Over the past two decades, the surgical management of renal masses has undergone a fundamental surgical shift. While radical nephrectomy (RN) was historically established as the gold standard for all renal tumors, partial nephrectomy (PN) has supplanted it as the preferred treatment for clinical T1 masses. This transition is supported by high-quality evidence demonstrating that nephron-sparing surgery offers cancer-specific survival equivalent to RN while providing superior preservation of renal function and potentially reducing cardiovascular morbidity [2, 3].

However, as surgical expertise has matured and robotic platforms have improved, the indication for partial nephrectomy has expanded beyond small tumors to include more complex cases. The management of pathologic T3a (pT3a) RCC—defined by tumor extension into the renal vein, perinephric fat, or renal sinus fat—remains one of the most contentious areas in urologic surgery today [4].

The critical issue facing clinicians is the profound absence of high-level evidence guiding the surgical approach to pT3a disease [5]. Current guidelines from major organizations, including the European Association of Urology (EAU) and the American Urological Association (AUA), acknowledge the technical feasibility of PN in selected T3a cases but stop short of a strong recommendation due to the scarcity of robust comparative data [6]. Consequently, the choice between partial and radical nephrectomy for these patients is frequently driven by surgeon preference and experience rather than standardized protocols.

The existing data landscape is dominated by retrospective, observational series that are inherently vulnerable to significant selection bias. In many reported cohorts, patients selected for partial nephrectomy tend to have smaller tumors, more favorable anatomy, and better baseline performance status compared to those undergoing radical nephrectomy [7]. This “indication bias” creates a statistical distortion where partial nephrectomy often appears to yield superior oncological survival outcomes compared to radical nephrectomy—a finding that biologically likely reflects the lower risk profile of the patients selected for PN rather than the efficacy of the procedure itself [8]. The absence of randomized data makes it nearly impossible to fully adjust for these confounders, leaving a gap in our understanding of whether PN is truly oncologically equivalent to RN for high-risk pT3a lesions.

Furthermore, there is a distinct lack of data distinguishing between “suspected” clinical T3a (cT3a) disease and unexpected pT3a upstaging. A significant proportion of pT3a cases are clinically diagnosed as T1 or T2 and only identified as locally advanced upon final pathological review [9]. The oncological behavior of these upstaged tumors compared to those with grossly identifiable extra-renal extension is poorly characterized. Without granular data stratifying these subgroups, it remains unclear if the surgical approach should be modified based on preoperative imaging features or if the presence of pT3a features universally mandates a more aggressive resection [10].

Given the potential for significant renal functional benefit with PN, but the notable risk of local recurrence if oncological principles are compromised, the need for clarity is urgent. The current body of literature is fragmented. This systematic review and meta-analysis aims to address this absence of definitive data. By synthesizing the available comparative studies, we seek to overcome the limitations of individual small-volume series and determine whether partial nephrectomy represents a safe and effective alternative to radical nephrectomy for selected patients with pT3a renal cell carcinoma.

## Methods

### Search strategy

This systematic review and meta-analysis adhered to the PRISMA 2020 guidelines. The study protocol was registered in PROSPERO (CRD420251044787). A comprehensive literature search was conducted in PubMed, Web of Science and Scopus databases up to July 2025 without language restrictions. Search terms combined controlled vocabulary and keywords related to “renal cell carcinoma,” “partial nephrectomy,” “radical nephrectomy,” and “pT3a”. Additional references were identified through screening of cited articles, conference abstracts, and trial registries.

The full search strategy is resumed in the Supplementary Table 1.

### Eligibility criteria

We included studies which were in accordance with the following PICOS criteria:

- **Population**: Adult patients (> 18 years) diagnosed with pathologic T3a (pT3a) renal cell carcinoma, analyzing at minimum 5 patients in the manuscript.
- **Intervention**: PN, performed open, laparoscopic, or robot-assisted.
- **Comparison**: RN, performed open, laparoscopic, or robot-assisted.
- **Outcomes**: At least one oncological outcome (Cancer-Specific Survival [CSS], Overall Survival [OS], Recurrence-Free Survival [RFS]) or perioperative/functional outcome (complications, estimated glomerular filtration rate [eGFR] change).
- **Study Design**: Randomized controlled trials (RCTs), prospective observational studies, or retrospective cohort studies, or case series of at least 5 patients.

Exclusion criteria were tumor stages other than pT3a, metastatic disease at diagnosis, prior neoadjuvant or adjuvant treatments, and insufficient follow-up (< 6 months).

### Study selection and data extraction

Two independent reviewers (F.R. and D.F.) screened titles and abstracts, followed by full-text assessment. A third independent reviewer was involved to resolve disagreements (F.G.). Data extraction was performed independently using a predefined form and included demographic, clinical, surgical, and outcome data. Missing or unclear data were not requested from authors.

### Outcomes

Primary outcomes were overall survival (OS), cancer-specific survival (CSS), and recurrence-free survival (RFS). Secondary outcomes included postoperative renal function (estimated glomerular filtration rate [eGFR], chronic kidney disease incidence, dialysis requirement), postoperative complications rate (according to Clavien Dindo [CD] classification), operative time, blood loss, hospital length of stay (LOS), positive surgical margins (PSM) rate, and readmission rate. Mean and standard deviation (SD) are used in case of quantitative variables, while absolute numbers and frequencies of events are used in case of qualitative variables.

### Statistical analysis

Meta-analyses were conducted using a random-effects model (DerSimonian–Laird method). Effect estimates were expressed as hazard ratios (HRs), odds ratios (ORs), or mean differences (MDs) with 95% confidence intervals (CIs). Heterogeneity was assessed with the I^2^ statistic. Sensitivity and subgroup analyses were performed when applicable. All analyses were conducted with STATA software version 19.0 (StataCorp, College Station, TX, USA), with the search date cutoff set in July 2025. Statistical significance was defined as *p* < 0.05.

### Risk of bias assessment

Risk of bias was independently evaluated by two reviewers (F.R. and D.F.). ROBINS-I for interventional studies. A third reviewer (F.G.) resolved discrepancies.

## Results

### Study characteristics

PRISMA flow chart diagram was produced to resume the identification, screening, and inclusion process (Supplementary Fig. 1). In total, 2038 papers were initially identified using the abovementioned databases. After an accurate screening phase, 16 papers were included in the present systematic review and meta-analysis [11–26]. All of them are retrospective studies, analyzing and comparing outcomes between PN and RN.

Overall, 34,304 patients were included, 5878 (17.1%) underwent PN and 28,426 (82.9%) underwent RN.

### Risk of bias

A Risk of Bias assessment was performed to define the risk of biases for each study included in the review. 8 papers showed a high risk of bias [11–13, 16, 18, 19, 22, 23], while other 8 papers showed moderate risk of bias [14, 15, 17, 20, 21, 24–26]. Results are shown in Supplementary Fig. 2 and Supplementary Fig. 3.

### Baseline characteristics

Characteristics at baseline are shown in Table 1. Regarding the PN group, mean age was 61.9 (3.8) years, female patients were 1223 (26.6%) in 14 studies [11–21, 23, 25, 26], mean Body Mass Index (BMI) was 28.9 (2.7) kg/m^2^, mean preoperative eGFR was 74.5 (2.7) mL/min, mean RENAL score was 8.8 (0.9) points.

**Table 1:** Baseline characteristics

Author, year	Country	Study design	PN patients *N*, %	RN patients *N*, %	PN Age [years] Mean (SD)	RN Age [years] Mean (SD)	PN Female *N*, %	RN Female *N*, %	PN BMI [kg/m^2^] Mean (SD)	RN BMI [kg/m^2^] Mean (SD)	PN RENAL score Mean (SD)	RN RENAL score Mean (SD)	PN preoperative eGFR [ml/min] Mean (SD)	RN preoperative eGFR [ml/min] Mean (SD)
Jeong S et al., 2016 [11]	South Korea	R	37 (40.7)	54 (59.3)	54.9 (15)	61.1 (12.7)	11 (29.7)	12 (22.2)	25 (2.9)	24.5 (3.2)				
Alesker A et al., 2023 [12]	Saudi Arabia	R	17 (11.6)	130 (88.4)	53 (9.7)	64.2 (17.2)	8 (47.1)	49 (37.7)	32.2 (7.1)	28.9 (6.1)				
Alvim R et al., 2021 [13]	USA	R	220 (37)	369 (63)	63 (11.9)	61.3 (11.9)	63 (29)	110 (30)					71 (20.6)	71.4 (20.6)
Hamilton Z et al., 2019 [14]	USA	R	120 (33.3)	240 (66.7)	60.5 (12.5)	60.9 (12.6)	36 (30)	71 (29.6)						
Hansen J et al., 2012 [15]	Canada	R	477 (9.1)	4755 (90.9)	64 (12.6)	64 (12.6)	114 (24)	1461 (31)						
Liu S et al., 2021 [16]	China	R	872 (27.3)	2324 (72.7)	63.6 (11.1)	65.3 (11.9)	236 (27.1)	760 (32.7)						
Liu Z et al., 2023 [17]	China	R	200 (15.7)	1077 (84.3)	61.9 (11.5)	63.3 (11.8)	57 (28.5)	326 (30.3)						
Muhlbauer et al., 2020 [18]	Germany	R	48 (30.4)	110 (69.6)	68.1 (13)	65 (12.8)	11 (22.9)	32 (29.1)	26.9 (4.6)	26.8 (4.5)	8 (1.8)	10 (0.9)	68 (17.8)	69.7 (24.5)
Patel SH et al., 2020 [19]	USA	R	243 (26.2)	686 (73.8)	63.2 (11.2)	62.9 (12.6)	59 (24.3)	202 (29.4)	29.2 (6.1)	27.3 (5.7)	9.9 (1.6)	8.8 (1.9)	70.2 (27.1)	74.9 (13.1)
Pecoraro A et al., 2022 [20]	Italy		1795 (13.6)	11,382 (86.4)	63.1 (0.2)	64.1 (0.1)	494 (27.5)	3512 (30.9)						
Saitta C et al., 2025 [21]	Italy	R	313 (28.2)	799 (71.8)	62.4 (4.5)	64.4 (11.1)	63 (20.1)	229 (28.7)	28.3 (5.6)	27.5 (5.3)	9 (1.5)	8.8 (1.7)	82.7 (24.4)	76.2 (23.8)
Ziegelmuller B et al., 2019 [22]	Germany	R	38 (69.1)	17 (30.9)										
Weight CJ et al., 2010 [23]	USA	R	96 (45.1)	117 (54.9)	63.4 (11.2)	67 (13.5)	23 (24)	42 (36)						
Tian J et al., 2022 [24]	China	R	1237 (17.4)	5890 (82.6)	63.8 (11.1)	63.8 (11.1)								
Andrade H et al., 2017 [25]	USA	R	70 (50)	70 (50)	62 (10.9)	62.7 (10.9)	19 (27.1)	22 (31.4)	31.5 (7)	29.5 (5.5)	8.1 (1.2)	7.9 (1.1)	80.8 (30.2)	75.6 (15.8)
Patel P et al., 2017 [26]	Canada	R	95 (19)	406 (81)			29 (30.5)	122 (30)						

USA, United States of America; R, Retrospective; P, Prospectical; PN, Partial Nephrectomy; RN, Radical Nephrectomy; SD, Standard Deviation; BMI, Body Mass Index; eGFR, estimated Glomerular Filtration Rate

Preoperative clinical Tumor (cT) classification is shown in Table 2. Only 7 studies [11, 15, 18, 19, 21, 23, 25] provided the clinical local classification according to tumor size and location. cT1 were 677 (52.7%), cT2 were 302 (23.5%), and cT3a were 305 (23.8%).

**Table 2:** Clinical TNM characteristics

Author, year	PN patients *N*, %	RN patients *N*, %	PN cT1 *N*, %	RN cT1 *N*, %	PN cT1a *N*, %	RN cT1a *N*, %	PN cT1b *N*, %	RN cT1b *N*, %	PN cT2 *N*, %	RN cT2 *N*, %	PN cT2a *N*, %	RN cT2a *N*, %	PN cT2b *N*, %	RN cT2b *N*, %	PN cT3 *N*, %	RN cT3 *N*, %
Jeong S et al., 2016 [11]	37 (40.7)	54 (59.3)	37 (100)	54 (100)	28 (75.7)	20 (37)	9 (24.3)	34 (63)	0 (0)	0 (0)	0 (0)	0 (0)	0 (0)	0 (0)	0 (0)	0 (0)
Alesker A et al., 2023 [12]	17 (11.6)	130 (88.4)														
Alvim R et al., 2021 [13]	220 (37)	369 (63)														
Hamilton Z et al., 2019 [14]	120 (33.3)	240 (66.7)														
Hansen J et al., 2012 [15]	477 (9.1)	4755 (90.9)	318 (67)	2790 (59)	60 (13)	526 (11)	258 (54)	2264 (48)	159 (34)	1965 (41)	141 (30)	1578 (33)	18 (4)	387 (8)	0 (0)	0 (0)
Liu S et al., 2021 [16]	872 (27.3)	2324 (72.7)														
Liu Z et al., 2023 [17]	200 (15.7)	1077 (84.3)														
Muhlbauer et al., 2020 [18]	48 (30.4)	110 (69.6)	0 (0)	0 (0)	0 (0)	0 (0)	0 (0)	0 (0)	19 (39.6)	49 (44.5)	17 (35.4)	34 (30.9)	2 (4.2)	15 (13.6)	0 (0)	61 (55.5)
Patel SH et al., 2020 [19]	243 (26.2)	686 (73.8)	151 (62.2)	352 (52.7)					92 (37.8)	334 (47.3)					0 (0)	0 (0)
Pecoraro A et al., 2022 [20]	1795 (13.6)	11,382 (86.4)														
Saitta C et al., 2025 [21]	313 (28.2)	799 (71.8)	140 (44.7)	332 (41.6)	74 (23.6)	74 (9.3)	66 (21.1)	258 (32.3)	20 (6.4)	239 (29.9)	18 (5.8)	154 (19.3)	2 (0.6)	85 (10.6)	153 (48.9)	228 (28.5)
Ziegelmuller B et al., 2019 [22]	38 (69.1)	17 (30.9)														
Weight CJ et al., 2010 [23]	96 (45.1)	117 (54.9)	0 (0)	0 (0)	0 (0)	0 (0)	0 (0)	0 (0)	12 (13)	23 (20)					84 (87)	94 (80)
Tian J et al., 2022 [24]	1237 (17.4)	5890 (82.6)														
Andrade H et al., 2017 [25]	70 (50)	70 (50)	31 (44.3)	27 (38.6)	23 (32.9)	7 (10)	8 (11.4)	20 (28.6)	0 (0)	0 (0)	0 (0)	0 (0)	0 (0)	0 (0)	39 (55.7)	43 (61.4)
Patel P et al., 2017 [26]	95 (19)	406 (81)														

PN, Partial Nephrectomy; RN, Radical Nephrectomy

Regarding the RN group, mean age was 63.6 (1.7) years, female patients were 6950 (30.9%) in 14 studies [11–21, 23, 25, 26], mean BMI was 27.4 (1.8) kg/m^2^, mean preoperative eGFR was 73.6 (2.9) mL/min, mean RENAL score was 8.9 (0.9) points.

Regarding preoperative cT classification, cT1 were 3555 (53.9%), cT2 were 2610 (39.6%), and cT3a were 426 (6.5%).

### Perioperative outcomes

Perioperative outcomes are shown in Table 3. Regarding the PN group, mean operative time was 173.3 (41.6) minutes, mean EBL was 266.6 (58.5) mL, mean WIT was 18.7 (6.7) minutes, a mininvasive (laparoscopic or robot-assisted) approach was used in 337 (55.8%) patients on 3 studies [18, 19, 21], 31 (3.6%) patients experienced a major (CD > 2) postoperative complication on 5 studies [13, 18, 19, 21, 22], 80 (7.3%) patients underwent a lymph node (LN) dissection on 2 studies [13, 16], and 67 (12.1%) patients underwent at least one transfusion on 2 studies [19, 21].

**Table 3:** Perioperative outcomes

Author, year	PN patients *N*, %	RN patients *N*, %	PN Operative time [min] Mean (SD)	RN Operative time [min] Mean (SD)	PN EBL [ml] Mean (SD)	RN EBL [ml] Mean (SD)	PN WIT [min] Mean (SD)	RN WIT [min] Mean (SD)	PN mininvasive technique *N*, %	RN mininvasive technique *N*, %	PN CD > 2 *N*, %	RN CD > 2 *N*, %	PN LN dissection *N*, %	RN LN dissection *N*, %	PN Transfusion *N*, %	RN Transfusion *N*, %
Jeong S et al., 2016 [11]	37 (40.7)	54 (59.3)														
Alesker A et al., 2023 [12]	17 (11.6)	130 (88.4)														
Alvim R et al., 2021 [13]	220 (37)	369 (63)			316.7 (261.2)	316.7 (260.5)					11 (5)	12 (3.3)	44 (20)	286 (78)		
Hamilton Z et al., 2019 [14]	120 (33.3)	240 (66.7)														
Hansen J et al., 2012 [15]	477 (9.1)	4755 (90.9)														
Liu S et al., 2021 [16]	872 (27.3)	2324 (72.7)											36 (4.1)	319 (13.7)		
Liu Z et al., 2023 [17]	200 (15.7)	1077 (84.3)														
Muhlbauer et al., 2020 [18]	48 (30.4)	110 (69.6)	143.8 (48)	151.5 (59.3)	335.4 (229.3)	250 (150.2)	20.7 (9.2)	0 (0)	2 (4.2)	13 (11.8)	4 (8.3)	14 (12.7)				
Patel SH et al., 2020 [19]	243 (26.2)	686 (73.8)	202.7 (81.3)	179.8 (74.3)	257.3 (154.3)	264.1 (216.6)	24.2 (21.8)	0 (0)	151 (62.2)	295 (43)	7 (2.9)	19 (2.8)			32 (13.2)	216 (31.5)
Pecoraro A et al., 2022 [20]	1795 (13.6)	11,382 (86.4)														
Saitta C et al., 2025 [21]	313 (28.2)	799 (71.8)			199.1 (149)	197.7 (151.5)			184 (58.5)	669 (83.7)	9 (2.9)	17 (2.1)			35 (11.2)	149 (18.6)
Ziegelmuller B et al., 2019 [22]	38 (69.1)	17 (30.9)			224.4 (385.2)	481 (970.1)	11.3 (19.3)	0 (0)			0 (0)	0 (0)				
Weight CJ et al., 2010 [23]	96 (45.1)	117 (54.9)														
Tian J et al., 2022 [24]	1237 (17.4)	5890 (82.6)														
Andrade H et al., 2017 [25]	70 (50)	70 (50)														
Patel P et al., 2017 [26]	95 (19)	406 (81)														

PN, Partial Nephrectomy; RN, Radical Nephrectomy; SD, Standard Deviation; EBL, Estimated Blood Loss; WIT, Warm Ischemia Time; CD, Clavien Dindo; LN, Lymph Node

Regarding the RN group, mean operative time was 165.7 (20) minutes, mean EBL was 301.9 (108.7) mL, a mininvasive (laparoscopic or robot-assisted) approach was used in 977 (61.3%) patients on 3 studies [18, 19, 21], 62 (3.1%) patients experienced a major (CD > 2) postoperative complication on 5 studies [13, 18, 19, 21, 22], 605 (22.5%) patients underwent a lymph node (LN) dissection on 2 studies [13, 16], and 365 (24.6%) patients underwent at least one transfusion on 2 studies [19, 21].

### Pathological outcomes

Pathological and oncological features are shown in Tables 4, 5, and 6.

**Table 4:** pT3a pathological invasion pattern

Author, year	PN patients *N*, %	RN patients *N*, %	PN Perirenal fat *N*, %	RN Perirenal fat *N*, %	PN Renal sinus *N*, %	RN Renal sinus *N*, %	PN Renal vein *N*, %	RN Renal vein *N*, %	PN Combined pattern *N*, %	RN Combined pattern *N*, %
Jeong S et al., 2016 [11]	37 (40.7)	54 (59.3)								
Alesker A et al., 2023 [12]	17 (11.6)	130 (88.4)								
Alvim R et al., 2021 [13]	220 (37)	369 (63)								
Hamilton Z et al., 2019 [14]	120 (33.3)	240 (66.7)								
Hansen J et al., 2012 [15]	477 (9.1)	4755 (90.9)								
Liu S et al., 2021 [16]	872 (27.3)	2324 (72.7)	683 (78.3)	1136 (48.9)	189 (21.7)	1188 (51.1)	0 (0)	0 (0)	0 (0)	0 (0)
Liu Z et al., 2023 [17]	200 (15.7)	1077 (84.3)	149 (74.5)	468 (43.5)	21 (11)	197 (18.3)	0 (0)	0 (0)	29 (14.5)	412 (38.2)
Muhlbauer et al., 2020 [18]	48 (30.4)	110 (69.6)								
Patel SH et al., 2020 [19]	243 (26.2)	686 (73.8)								
Pecoraro A et al., 2022 [20]	1795 (13.6)	11,382 (86.4)								
Saitta C et al., 2025 [21]	313 (28.2)	799 (71.8)								
Ziegelmuller B et al., 2019 [22]	38 (69.1)	17 (30.9)								
Weight CJ et al., 2010 [23]	96 (45.1)	117 (54.9)								
Tian J et al., 2022 [24]	1237 (17.4)	5890 (82.6)								
Andrade H et al., 2017 [25]	70 (50)	70 (50)	35 (50)	16 (22.9)					38 (54.3)	60 (85.7)
Patel P et al., 2017 [26]	95 (19)	406 (81)								

PN, Partial Nephrectomy; RN, Radical Nephrectomy

**Table 5:** pT3a Pathological grading features

Author, year	PN patients *N*, %	RN patients *N*, %	PN Fuhrman G1 *N*, %	RN Fuhrman G1 *N*, %	PN Fuhrman G2 *N*, %	RN Fuhrman G2 *N*, %	PN Fuhrman G3 *N*, %	RN Fuhrman G3 *N*, %	PN Fuhrman G4 *N*, %	RN Fuhrman G4 *N*, %	PN Fuhrman G undefined *N*, %	RN Fuhrman G undefined *N*, %
Jeong S et al., 2016 [11]	37 (40.7)	54 (59.3)	1 (2.7)	0 (0)	15 (40.5)	17 (31.5)	20 (54.1)	30 (55.6)	1 (2.7)	7 (13)	0 (0)	0 (0)
Alesker A et al., 2023 [12]	17 (11.6)	130 (88.4)										
Alvim R et al., 2021 [13]	220 (37)	369 (63)	0 (0)	1 (0.3)	47 (25)	35 (10)	104 (54)	172 (51)	23 (12)	115 (34)	17 (8.9)	13 (3.9)
Hamilton Z et al., 2019 [14]	120 (33.3)	240 (66.7)										
Hansen J et al., 2012 [15]	477 (9.1)	4755 (90.9)										
Liu S et al., 2021 [16]	872 (27.3)	2324 (72.7)										
Liu Z et al., 2023 [17]	200 (15.7)	1077 (84.3)	15 (7.5)	43 (4)	102 (51)	390 (36.2)	74 (37)	461 (42.8)	9 (4.5)	183 (17)	0 (0)	0 (0)
Muhlbauer et al., 2020 [18]	48 (30.4)	110 (69.6)										
Patel SH et al., 2020 [19]	243 (26.2)	686 (73.8)										
Pecoraro A et al., 2022 [20]	1795 (13.6)	11,382 (86.4)										
Saitta C et al., 2025 [21]	313 (28.2)	799 (71.8)										
Ziegelmuller B et al., 2019 [22]	38 (69.1)	17 (30.9)										
Weight CJ et al., 2010 [23]	96 (45.1)	117 (54.9)										
Tian J et al., 2022 [24]	1237 (17.4)	5890 (82.6)	68 (5.5)	166 (2.8)	508 (41.1)	1946 (33)	414 (33.5)	2303 (39.1)	73 (5.9)	763 (13)	174 (14.1)	712 (12.1)
Andrade H et al., 2017 [25]	70 (50)	70 (50)										
Patel P et al., 2017 [26]	95 (19)	406 (81)										

PN, Partial Nephrectomy; RN, Radical Nephrectomy

**Table 6:** pT3a Pathological histological features

Author, year	PN patients *N*, %	RN patients *N*, %	PN Clear Cell Carcinoma *N*, %	RN Clear Cell Carcinoma *N*, %	PN Papillary Carcinoma *N*, %	RN Papillary Carcinoma *N*, %	PN Chromophobe Carcinoma *N*, %	RN Chromophobe Carcinoma *N*, %	PN Other *N*, %	RN Other *N*, %	PN Size Mean (SD)	RN Size Mean (SD)	PN PSM N, %	RN PSM N, %
Jeong S et al., 2016 [11]	37 (40.7)	54 (59.3)	28 (75.7)	41 (75.9)	3 (8.1)	4 (7.4)	5 (13.5)	6 (11.1)	1 (2.7)	3 (5.6)			2 (5.4)	0 (0)
Alesker A et al., 2023 [12]	17 (11.6)	130 (88.4)												
Alvim R et al., 2021 [13]	220 (37)	369 (63)	170 (77)	323 (87.5)	21 (10)	13 (3.5)	29 (13)	33 (9)	0 (0)	0 (0)	4 (1.5)	8.3 (3.7)	19 (8.6)	12 (3.3)
Hamilton Z et al., 2019 [14]	120 (33.3)	240 (66.7)												
Hansen J et al., 2012 [15]	477 (9.1)	4755 (90.9)	354 (74)	4323 (91)	92 (19)	289 (6)	31 (7)	143 (3)	0 (0)	0 (0)	3.9 (3.2)	7.3 (6.5)		
Liu S et al., 2021 [16]	872 (27.3)	2324 (72.7)	523 (60)	1961 (72.8)	0 (0)	0 (0)	0 (0)	0 (0)	349 (40)	633 (27.2)	3.5 (1.4)	5.1 (1.5)		
Liu Z et al., 2023 [17]	200 (15.7)	1077 (84.3)	119 (59.5)	794 (73.7)	42 (21)	71 (6.6)	14 (7)	54 (5)	25 (12.5)	158 (14.7)				
Muhlbauer et al., 2020 [18]	48 (30.4)	110 (69.6)	28 (58.3)	92 (83.6)									2 (4.3)	5 (4.6)
Patel SH et al., 2020 [19]	243 (26.2)	686 (73.8)											15. 6.1	29. 4.2
Pecoraro A et al., 2022 [20]	1795 (13.6)	11,382 (86.4)	1178 (65.6)	9628 (84.6)	397 (22.1)	788 (6.9)	197 (11)	630 (5.5)	23 (1.3)	336 (2.9)	4.2 (0.05)	7.8 (0.04)		
Saitta C et al., 2025 [21]	313 (28.2)	799 (71.8)	124 (39.6)	285 (35.7)							4.4 (0.8)	7.2 (3)	36 (11.5)	21 (2.6)
Ziegelmuller B et al., 2019 [22]	38 (69.1)	17 (30.9)									3.8 (4.2)	3.9 (4.9)		
Weight CJ et al., 2010 [23]	96 (45.1)	117 (54.9)									3.9 (2)	5.9 (1.3)		
Tian J et al., 2022 [24]	1237 (17.4)	5890 (82.6)									4.1 (1.8)	7.5 (3)		
Andrade H et al., 2017 [25]	70 (50)	70 (50)	50 (71.4)	64 (91.4)							4 (1.7)	4.7 (1.1)	4 (5.7)	4 (5.7)
Patel P et al., 2017 [26]	95 (19)	406 (81)											14 (14.7)	32 (7.9)

PN, Partial Nephrectomy; RN, Radical Nephrectomy; SD, Standard Deviation; PSM, Positive Surgical Margins

Only 4 studies [16–18, 25] evaluated the invasion pattern of pT3a tumors. In the PN group, 894 (75.6%) showed a perirenal fat invasion, 210 (17.8%) showed a renal sinus involvement, 0 (0%) showed an involvement of the renal vein, and 67 (6.6%) showed a combined involvement of structures. In the RN group, 1665 (46.8%) showed a perirenal fat invasion, 1388 (38.8%) showed a renal sinus involvement, 26 (0.2%) showed an involvement of the renal vein, and 472 (13.5%) showed a combined involvement of structures.

4 studies [11, 13, 17, 24] evaluated the pathological grading, according to Fuhrman’s grading classification. In the PN group, 84 (5.4%) patients had a Grade (G) 1 disease, 672 (39.9%) patients had a G2 disease, 612 (36.5%) patients had a G3 disease, 106 (6.4%) patients had a G4 disease, and in 191 (11.8%) cases the grading was not reported. In the RN group, 210 (2.9%) patients had a G1 disease, 2388 (32.4%) patients had a G2 disease, 2966 (40.3%) patients had a G3 disease, 1068 (14.6%) patients had a G4 disease, and in 725 (9.8%) cases the grading was not reported.

Regarding tumor histology, in the PN group, 2574 (63.8%) masses were clear cell carcinomas on 9 studies [11, 13, 15–18, 20, 21, 25], 555 (15.4%) were papillary carcinomas on 6 studies [11, 13, 15–17, 20], 276 (7.7%) were chromophobe carcinomas on 6 studies [11, 13, 15–17, 20], and 398 (11.1%) were other histological tumors on 6 studies [11, 13, 15–17, 20]. In the RN group, 17,241 (82.3%) masses were clear cell carcinomas on 9 studies [11, 13, 15–18, 20, 21, 25], 1165 (5.8%) were papillary carcinomas on 6 studies [11, 13, 15–17, 20], 866 (4.3%) were chromophobe carcinomas on 6 studies [11, 13, 15–17, 20], and 1130 (5.7%) were other histological tumors on 6 studies [11, 13, 15–17, 20]. Mean pathological size in the PN group was 4 (0.3) cm and PSM rate was 92 (9%) on 7 studies [11, 13, 18, 19, 21, 25, 26]. In the RN group, mean pathological size was 6.4 (1.6) cm and PSM rate was 103 (4.1%) on 7 studies [11, 13, 18, 19, 21, 25, 26].

### Functional and oncological outcomes

Functional and oncological outcomes are shown in Table 7. In the PN group, mean postoperative eGFR was 62.5 (2.1) ml/min, mean LOS was 4 (1.4), recurrence rate was 97 (14.2%) on 5 studies [12, 19, 21, 22, 25], metastasis (M) progression rate was 7 (5.6%) on 3 studies [12, 22, 25], CSS rate at 5 years was 1394 (95.8%) on 4 studies [16, 17, 21, 25], and OS rate at 5 years was 1271 (87.4%) on 4 studies [16, 17, 21, 25].

**Table 7:** Postoperative outcomes

Author, year	PN patients *N*, %	RN patients *N*, %	PN postoperative eGFR Mean (SD)	RN postoperative eGFR Mean (SD)	PN LOS Mean (SD)	RN LOS Mean (SD)	PN Recurrence *N*, %	RN Recurrence *N*, %	PN M progression *N*, %	RN M progression *N*, %	PN CSS 5 years *N*, %	RN CSS 5 years *N*, %	PN OS 5 years *N*, %	RN OS 5 years *N*, %
Jeong S et al., 2016 [11]	37 (40.7)	54 (59.3)												
Alesker A et al., 2023 [12]	17 (11.6)	130 (88.4)					0 (0)	8 (6.4)	0 (0)	21 (16.8)				
Alvim R et al., 2021 [13]	220 (37)	369 (63)	63.5 (23.7)	53.2 (14.9)	3 (1.5)	2.3 (0.7)								
Hamilton Z et al., 2019 [14]	120 (33.3)	240 (66.7)												
Hansen J et al., 2012 [15]	477 (9.1)	4755 (90.9)												
Liu S et al., 2021 [16]	872 (27.3)	2324 (72.7)									837 (96)	2050 (88.2)	746 (85.5)	1762 (75.8)
Liu Z et al., 2023 [17]	200 (15.7)	1077 (84.3)									185 (92.6)	816 (75.8)	172 (85.9)	730 (67.8)
Muhlbauer et al., 2020 [18]	48 (30.4)	110 (69.6)	60.1 (21.8)	47.4 (15)										
Patel SH et al., 2020 [19]	243 (26.2)	686 (73.8)	63.9 (26.6)	55.5 (24.8)	5 (2.8)	7.7 (4.8)	64 (26.3)	204 (29.7)						
Pecoraro A et al., 2022 [20]	1795 (13.6)	11,382 (86.4)												
Saitta C et al., 2025 [21]	313 (28.2)	799 (71.8)					29 (9.3)	200 (25)			304 (97.1)	698 (87.4)	287 (91.7)	628 (78.6)
Ziegelmuller B et al., 2019 [22]	38 (69.1)	17 (30.9)					2 (5.3)	2 (11.8)	1 (2.6)	0 (0)				
Weight CJ et al., 2010 [23]	96 (45.1)	117 (54.9)												
Tian J et al., 2022 [24]	1237 (17.4)	5890 (82.6)												
Andrade H et al., 2017 [25]	70 (50)	70 (50)					2 (2.9)	1 (1.4)	6 (8.6)	4 (5.7)	68 (97.1)	68 (97.1)	66 (94.3)	61 (87.1)
Patel P et al., 2017 [26]	95 (19)	406 (81)												

PN, Partial Nephrectomy; RN, Radical Nephrectomy; SD, Standard Deviation; eGFR, estimated Glomerular Filtration Rate; LOS, length of stay; M, Metastasis; CSS, Cancer Specific Survival; OS, Overall Survival

In the RN group, mean postoperative eGFR was 52 (4.2) ml/min, mean LOS was 5 (3.8) days, recurrence rate was 415 (24.4%) on 5 studies [12, 19, 21, 22, 25], metastasis (M) progression rate was 25 (11.5%) on 3 studies [12, 22, 25], CSS rate at 5 years was 3632 (85.1%) on 4 studies [16, 17, 21, 25], and OS rate at 5 years was 3181 (74.5%) on 4 studies [16, 17, 21, 25].

### Sensitivity analysis

**Fig. 1:**
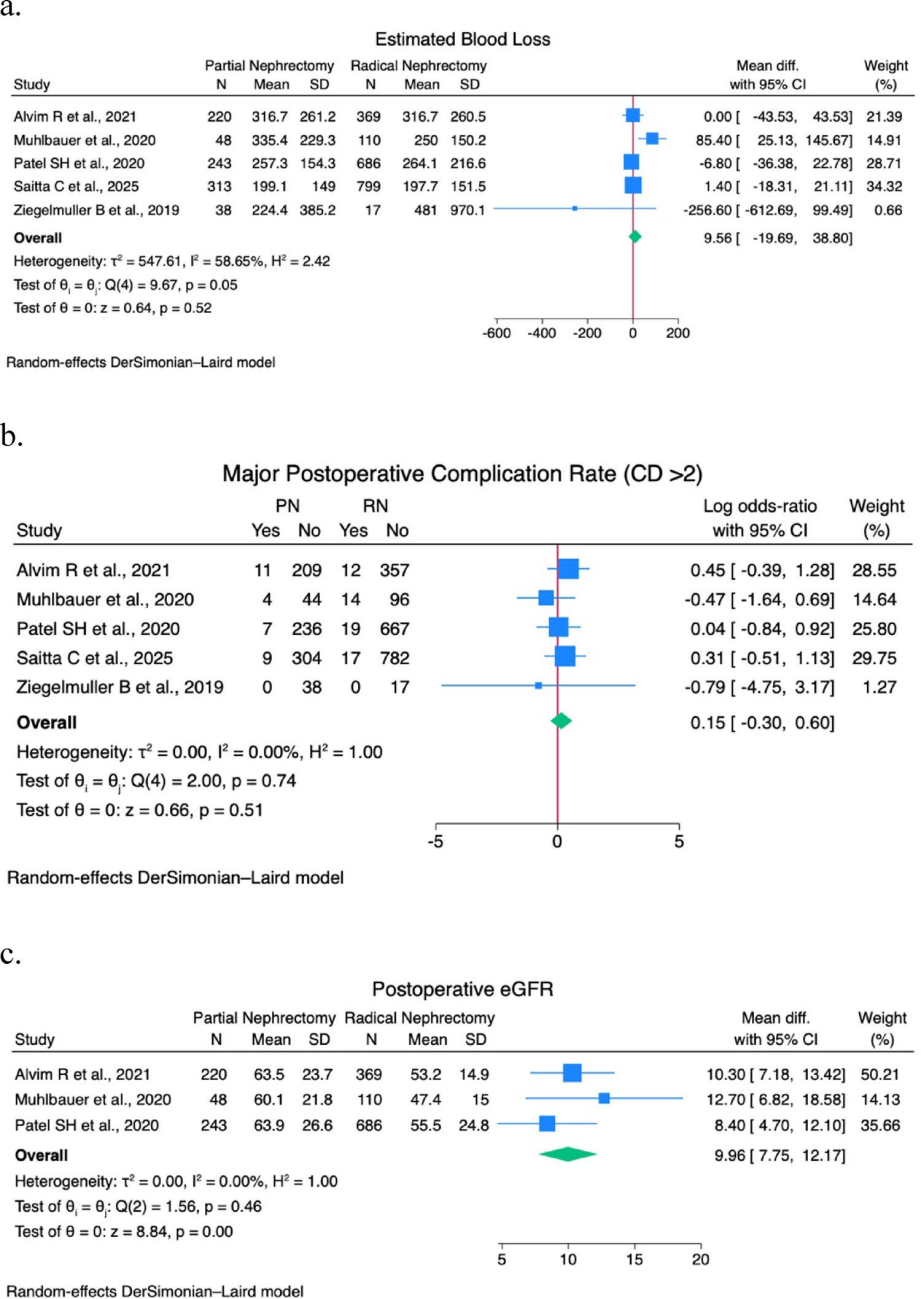

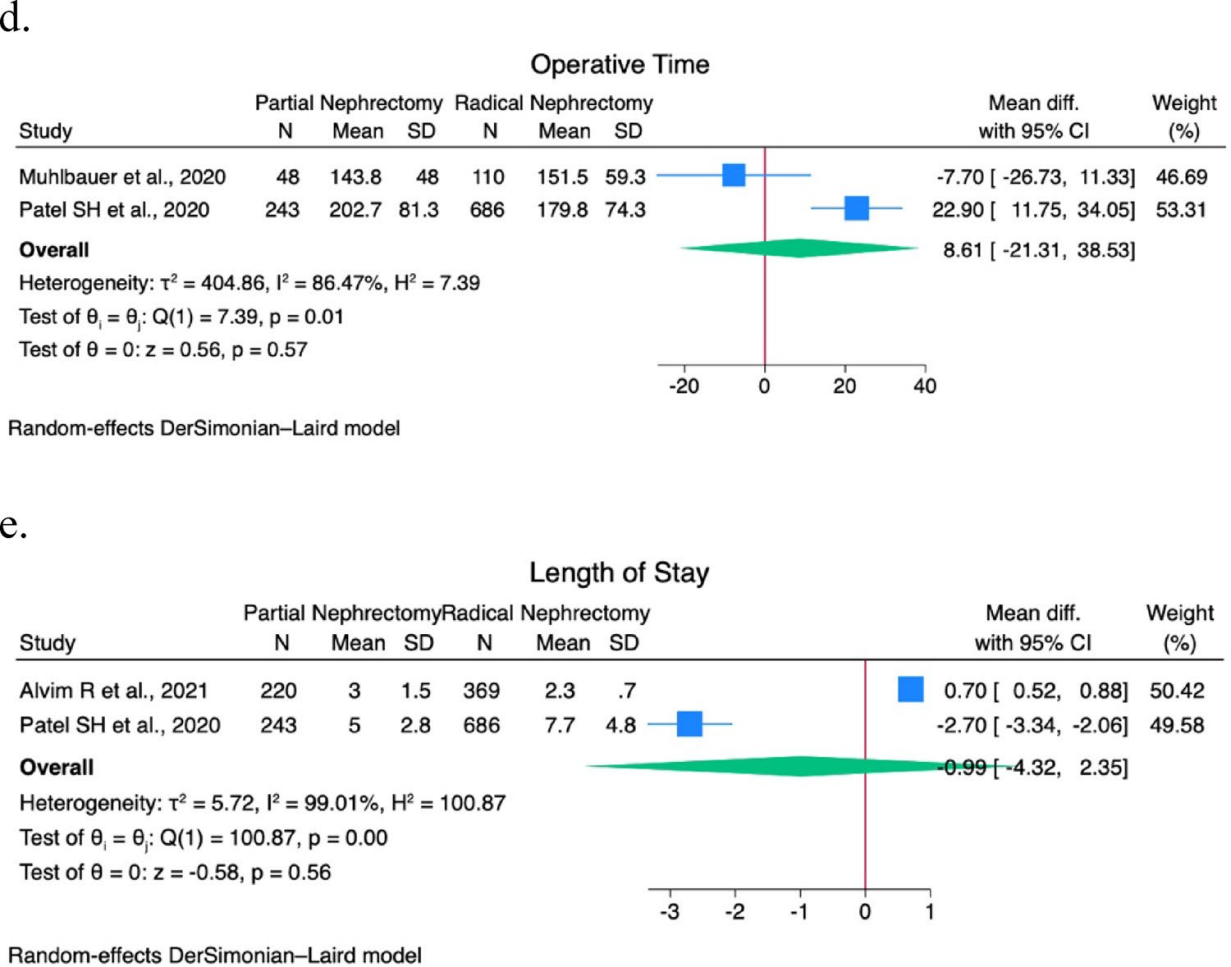
(**a**) Estimated blood loss forest plot; (**b**) Major postoperative complications (CD>2) rate forest plot; (**c**) Postoperative eGFR forest plot; (**d**) Operative time forest plot; (**e**) Length of stay forest plot

**Fig. 2:**
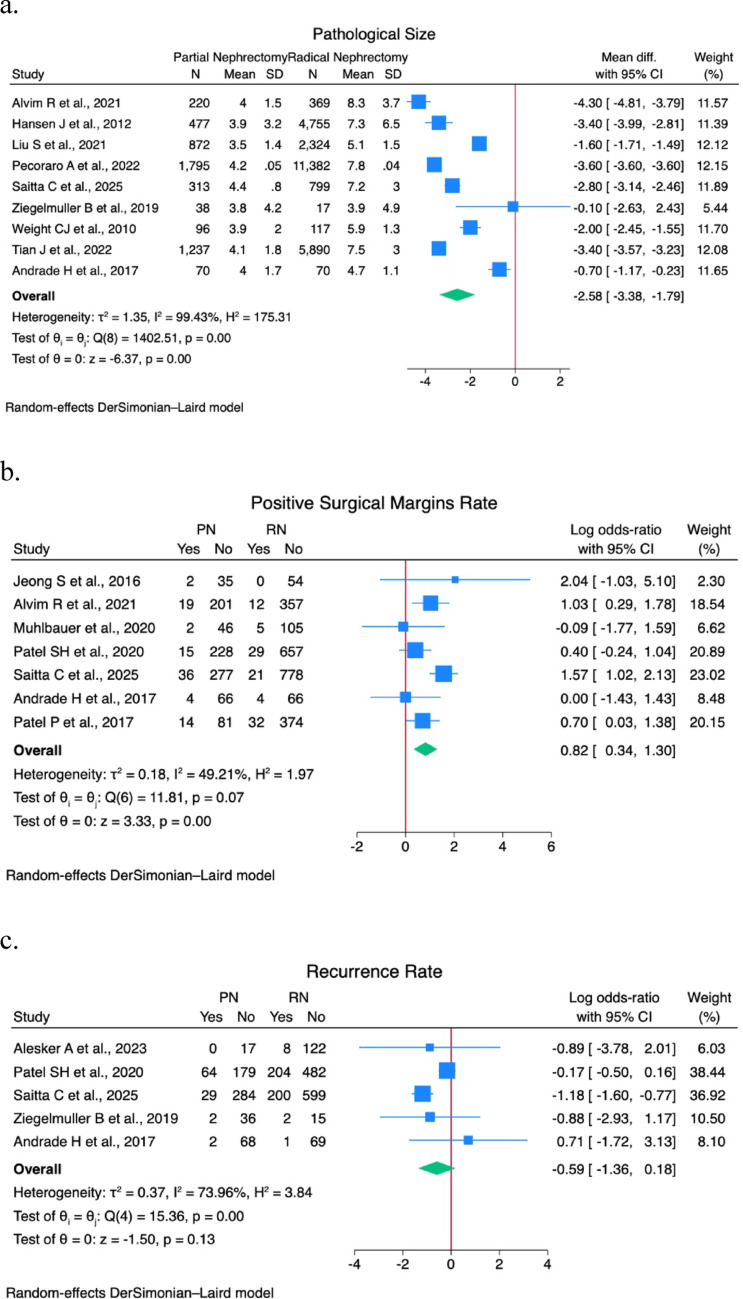

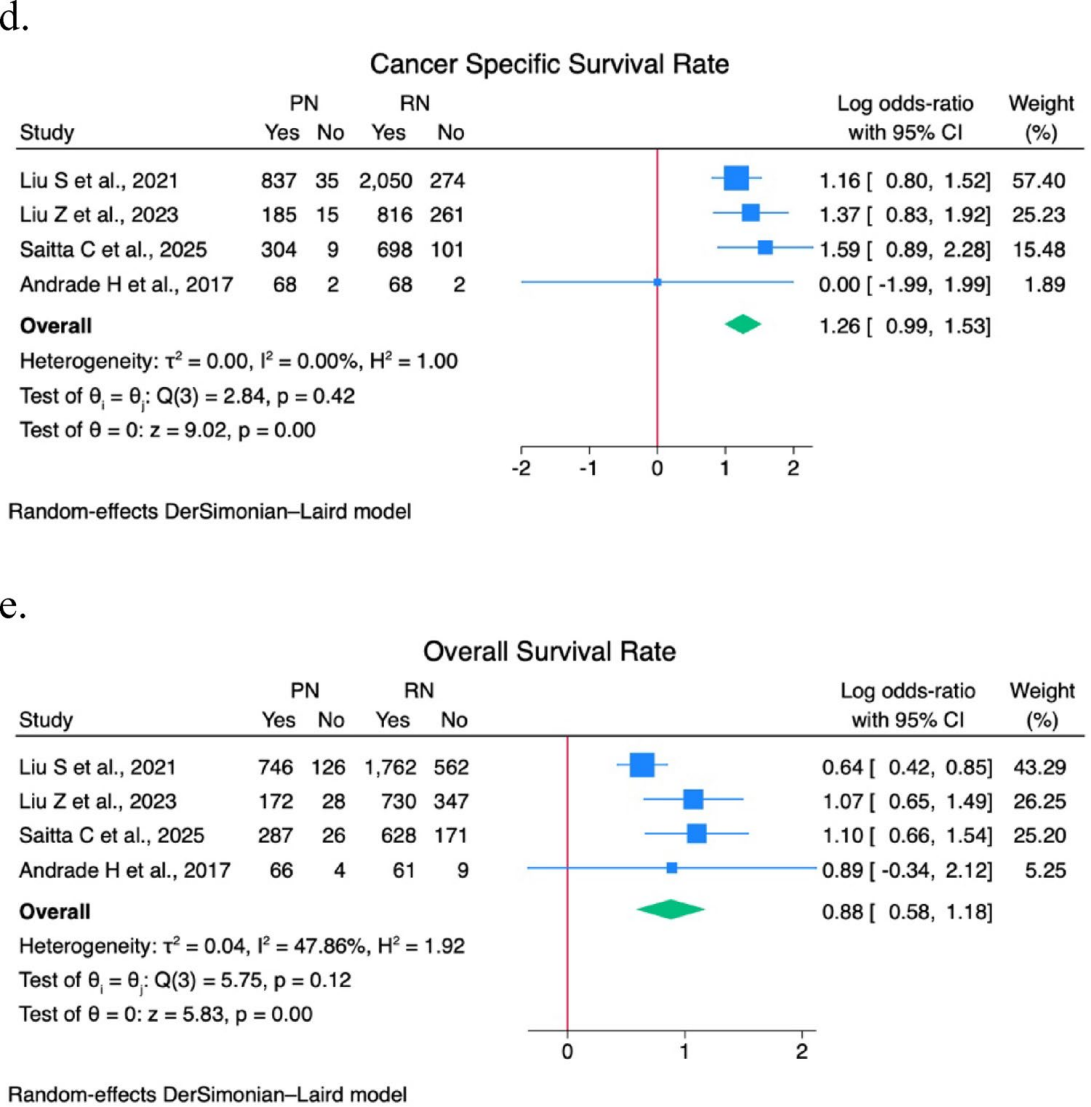
(**a**) Pathological tumor size forest plot; (**b**) Positive surgical margins rate forest plot; (**c**) Recurrence rate forest plot; (**d**) Cancer specific survival rate forest plot; (**e**) Overall survival rate forest plot

A sensitivity analysis was performed on EBL, operative time, postoperative eGFR, LOS, pathological size, major postoperative complications (CD > 2) rate, PSM rate, recurrence rate, CSS rate, and OS rate. Forest plots are shown in Figs. 1 and 2.

No statistically significant difference was found in EBL (9.56; 95% CI -19.7–38.8; I^2^ 58.7%; *p* = 0.52), operative time (8.61; 95% CI -21.3–38.5; I^2^ 86.5%; *p* = 0.57), and LOS (-0.99; 95% CI -4.3–2.4; I^2^ 99%; *p* = 0.56), CD > 2 complications rate (0.15; 95% CI -0.3–0.6; I^2^ 0%; *p* = 0.51), and recurrence rate (-0.59; 95% CI -1.4–0.2; I^2^ 74%; *p* = 0.13).

A statistically significant difference was found in postoperative eGFR (9.96; 95% CI 7.8–12.2; I^2^ 0%; *p* < 0.01), pathological tumor size (-2.6; 95% CI -3.4 – -1.8; I^2^ 99.4%; *p* < 0.01), PSM rate (0.82; 95% CI 0.3–1.3; I^2^ 49.2%; *p* < 0.01), CSS rate (1.26; 95% CI 0.99–1.5; I^2^ 0%; *p* < 0.01), and OS rate (0.9; 95% CI 0.6–1.2; I^2^ 47.9%; *p* < 0.01).

## Discussion

Our systematic review and meta-analysis aimed to compare perioperative and postoperative outcomes between PN and RN. Our findings underscored that EBL, operative time, LOS, and the major complications (CD > 2) rate did not differ significantly between PN and RN, confirming that, in high‑volume centers, a more extensive resection does not translate into greater perioperative morbidity.

Our results showed a significant mean difference in terms of tumor size between PN and RN. Renal masses treated with RN are significantly larger than those treated with PN, as shown by the mean difference of about 2.5–3 cm in favor of PN, which highlights a certain selection bias toward more complex tumors in the RN arm. The abovementioned difference stands in the guidelines’ indication for PN, limited to cT1a and cT1b tumors [27]. However, in the last years, conservative procedures are considered feasible and safe in selected patients, thanks to minimally invasive and more precise procedures performed using robotic platforms [28, 29]. These promising results led to the topic of the present article.

The rate of positive surgical margins is higher after PN, but without an apparent negative impact on overall or cancer‑specific survival in the available follow‑up, in line with the literature on disease ≤T2 [5].

PN is associated with significantly better preservation of postoperative eGFR (mean difference ~ 10 ml/min/1.73 m²), confirming the nephroprotective advantage already demonstrated in unselected cohorts [30].

Better results emerge in terms of overall and cancer-specific survival with PN compared to RN, suggesting that, in appropriately selected patients, a nephron-sparing approach does not compromise and may even improve oncologic control in pT3a disease; however, due to the heterogeneity of the available studies and data, these findings should not be generalized.

Our results are consistent with previous studies conducted on pT1 and pT2 RCC, showing that PN provides oncologic outcomes comparable to RN, with a clear benefit in terms of renal function and superiority in overall survival [31, 32]. In the specific context of pT3a stage disease, the available data are limited, but some observational series and propensity‑matched analyses have suggested that, in selected patients with disease confined to the kidney or with minimal extrarenal extension, PN does not entail an increased risk of local or metastatic recurrence compared with RN [21, 23]. Our pooled estimates of OS and CSS, which shows significant differences between the two approaches in favor of PN, it supports the hypothesis that the biological burden of pT3a disease can be effectively controlled even with nephron‑sparing surgery, provided that an oncologically adequate resection is achieved.

The higher rate of positive margins after PN, already described also for T1b–T2 tumors, represents a critical element that must be weighed against the functional benefit. However, several studies indicate that, in the absence of gross residual disease, PSMs do not systematically translate into worse survival, but they do require closer follow‑up and careful counselling [33, 34].

Several limitations need to be acknowledged. Some of the studies included analyzed a consistent population, but an important selection bias occurred. Several studies, as can be seen in the risk of bias assessment, suffer from the absence of a population matching between PN group and RN group, leading to misleading results. Furthermore, several papers do not report the surgical technique, which limits subgroup analysis.

The current evidence represents initial findings in the comparison between the two procedures. Future studies, using paired and weighted baseline features, could give new information. Prospective and randomized studies with larger cohorts are needed to validate our findings and assess long-term oncological and functional outcomes.

High and significant heterogeneity can be found in the analysis of some outcomes, which may be attributed to the inclusion of various types of study designs. This variability could potentially impact the reliability of the meta-analysis. A potentially impactful publication bias and small study bias may be found, reducing statistical strength of our findings, but the low reliability of funnel plots could not help us in identification of more biased studies. Better built comparative studies are necessary to conclusively define the non-inferiority of PN when compared with RN in pT3a diseases.

## Conclusions

In conclusion, this systematic review and meta-analysis challenge the reluctance in performing PN for T3a RCC, indicating that it is a safe and feasible alternative for selected pT3a cases. PN offers better functional preservation of renal function without increasing perioperative morbidity, blood loss, or major complications. Despite a higher incidence of PSM, there were improvements in terms of CSS or OS compared to RN. However, limits underscore the urgent need for prospective randomized trials to definitively confirm its non-inferiority to RN.

## Supplementary Information

Below is the link to the electronic supplementary material.

Supplementary Material 1

## Data Availability

The data presented in this study are available on request from the corresponding author.
