# Supplementary material for: Partial versus radical nephrectomy for pT3a renal cell carcinoma: a systematic review and meta-analysis
Source: J Robot Surg. 2026 Apr 21;20(1):446. doi: 10.1007/s11701-026-03384-8 (PMC13095967; doi:10.1007/s11701-026-03384-8)
Supplement: Supplementary file 1 — Supplementary Material 1 [file 11701_2026_3384_MOESM1_ESM.docx]

**Supplementary Table 1.** Full search strategy

| PubMed: 650 results |
| --- |
| ("renal cancer"[All Fields] OR "kidney cancer"[All Fields] OR "kidney malignancy"[All Fields] OR "renal malignancy"[All Fields] OR "kidney tumor"[All Fields] OR "renal tumor"[All Fields] OR "renal tumour"[All Fields] OR "kidney tumour"[All Fields] OR "RCC"[All Fields] OR "renal cell tumor"[All Fields] OR "renal cell tumour"[All Fields] OR "renal cell cancer"[All Fields] OR "renal cell carcinoma"[All Fields] OR "kidney carcinoma"[All Fields]) AND ("nephrectomy"[All Fields] OR "partial nephrectomy"[All Fields] OR "total nephrectomy"[All Fields] OR "radical nephrectomy"[All Fields]) AND ("stage 3"[tiab] OR "T3a"[tiab] OR "T3"[tiab] OR "pT3a"[tiab]) |
| Web of Science: 575 results |
| TS=((“renal cancer" OR "kidney cancer" OR "kidney malignancy" OR "renal malignancy" OR "kidney tumor" OR "renal tumor" OR "renal tumour" OR "kidney tumour" OR "RCC" OR "renal cell tumor" OR "renal cell tumour" OR "renal cell cancer" OR "renal cell carcinoma" OR "kidney carcinoma") AND ("nephrectomy" OR "partial nephrectomy" OR "total nephrectomy" OR "radical nephrectomy") AND ("stage 3" OR "T3a" OR "T3" OR "pT3a")) |
| Scopus: 813 results |
| TITLE-ABS-KEY("renal cancer" OR "kidney cancer" OR "kidney malignancy" OR "renal malignancy" OR "kidney tumor" OR "renal tumor" OR "renal tumour" OR "kidney tumour" OR "RCC" OR "renal cell tumor" OR "renal cell tumour" OR "renal cell cancer" OR "renal cell carcinoma" OR "kidney carcinoma" AND "nephrectomy" OR "partial nephrectomy" OR "total nephrectomy" OR "radical nephrectomy" AND "stage 3" OR "T3a" OR "T3" OR "pT3a") |

**Identification of studies via databases and registers**

Records identified from:

PubMed (n=650)

Web Of Science (n=575)

Scopus (n=813)

Total (n=2038)

Records removed *before screening*:

Duplicate records removed (n=765)

**Identification**

Records excluded

Out of Scope (n=967)

Review (n=189)

Case report (n=26)

Editorial or Letter (n=57)

Records screened

(n=1273)

Reports sought for retrieval

(n=0)

Reports not retrieved (n=0)

**Screening**

Reports excluded:

Inconsistent data (n=10)

Already included in a wider study that provides more information or article more recently published (n=8)

Reports assessed for eligibility

(n=34)

Studies included in review

(n=16)

**Included**

**Supplementary Figure 1.** PRISMA flow diagram for the selection process


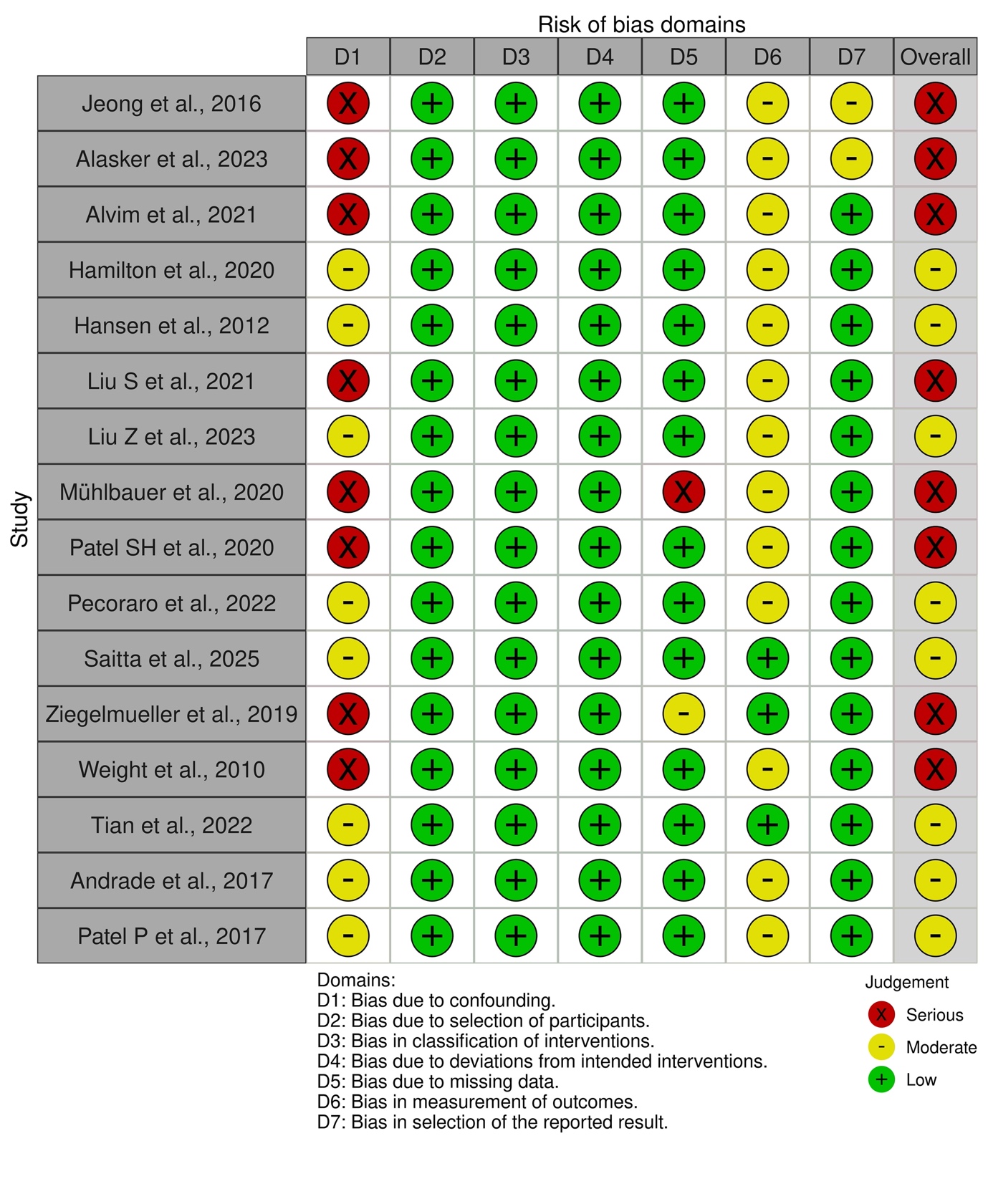


**Supplementary Figure 2.** Risk of Bias ROBINS-I traffic-light plot


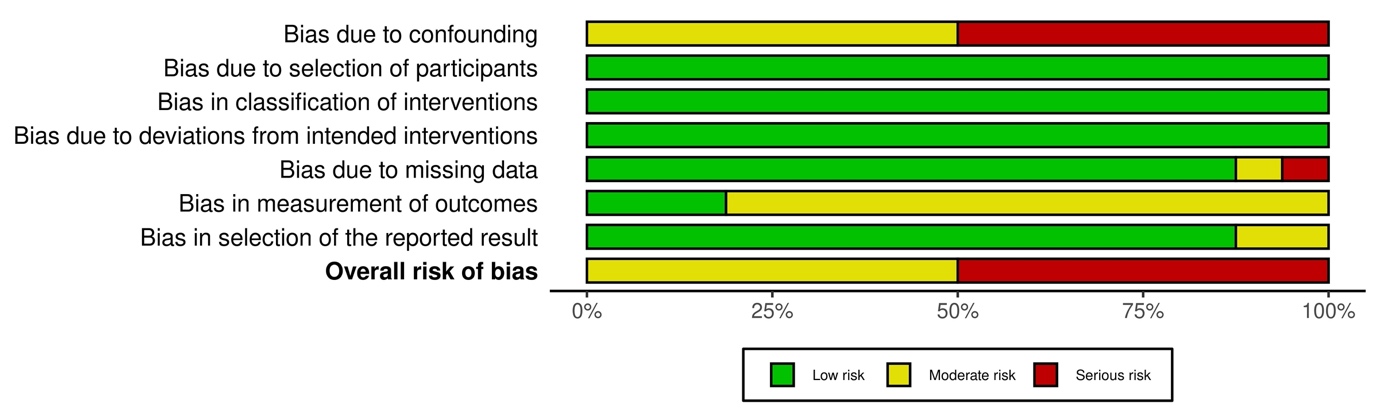


**Supplementary Figure 3.** Risk of Bias ROBINS-I summary plot
